# A Technology-Based Intervention to Reduce Alcohol Use After Metabolic and Bariatric Surgery: Examination of Six-month Follow-up Outcomes Following a Pilot Randomized Controlled Trial

**DOI:** 10.1007/s11695-026-08804-0

**Published:** 2026-06-12

**Authors:** Lisa R. Miller-Matero, Celeste Pappas, Brittany Christopher, Roman Grossi, Alyssa Vanderziel, Penelope J. Friday, Nancy P. Barnett, Roland S. Moore, Arthur M. Carlin, Oliver A. Varban, Jordan M. Braciszewski

**Affiliations:** 1 College of Human Medicine, Michigan State University, East Lansing, USA; 2 Henry Ford Health System, Detroit, USA; 3 Brown University, Providence, USA; 4 Pacific Institute For Research and Evaluation, Berkeley, USA

**Keywords:** Metabolic and bariatric surgery, Alcohol use, Technology-based intervention, Text messaging

## Abstract

**Background:**

Individuals who undergo metabolic and bariatric surgery (MBS) are at increased risk for an alcohol use disorder (AUD). Effective interventions are needed to mitigate this risk. The purpose of this study was to examine outcomes six months after participation in a randomized controlled trial of a technology-based intervention to reduce alcohol use after MBS.

**Methods:**

Patients between 3- and 18-months post-MBS were randomized to the intervention or treatment-as-usual control group. The intervention group completed two brief web-based sessions followed by three months of daily text messaging. Participants completed measures at baseline and at 6-month follow-up. Analyses were adjusted for baseline values.

**Results:**

There were 54 participants (90% retention) who completed the 6-month follow-up. Participants were primarily female (90.7%) and White (55.6%) or Black (38.9%), with a mean age of 44 years. At the 6-month follow-up, the intervention group had significantly lower odds of drinking compared to the control group (OR= 0.16, *p*= .04). The intervention group also consumed significantly fewer drinks per drinking day (F = 6.07, *p*= .02). There were no significant differences between the intervention and control groups for drinking frequency, ratings on the importance of avoiding alcohol use, or alcohol use disorder symptoms (*p*> .05).

**Conclusions:**

Findings suggest that a technology-based intervention for individuals who underwent MBS has the potential to lower alcohol use six months after the intervention concludes. The next step in this line of work is to conduct a fully powered trial to determine efficacy of reducing alcohol use and impact on AUD risk.

## Introduction

Metabolic and bariatric surgery (MBS) is the most effective treatment available for sustainable weight loss in patients with class II and III obesity and improvement of associated medical conditions [1–3]. Although there are many benefits of MBS, a subset of individuals may develop an alcohol use disorder (AUD), with as many as one in five individuals experiencing AUD following MBS [4]. Reasons for the increased risk of a postoperative AUD are still being explored, but likely include changes in the pharmacokinetics of alcohol and increases in the sensitivity and reward from alcohol use following MBS [5–7].

The risk of postoperative AUD is well known, and because of this, recommendations for the preoperative evaluation encourage a thorough assessment of alcohol use history and provision of education to patients regarding their increased risk [8]. Work has also been done to develop and pilot a more intensive preoperative program for individuals with a history of risky alcohol use, which has been successful at increasing knowledge about the risks of postoperative substance use [9]. However, despite patients recalling receiving education about the increased risk of a postoperative AUD, the majority of patients will consume alcohol after MBS [10]. Though education about risk remains an important approach, this suggests that additional methods are needed to mitigate risk of postoperative AUDs.

Our team previously developed and piloted a post-operative intervention aimed at reducing postoperative alcohol use. This intervention was developed and refined through feedback from patients who were consuming alcohol after MBS followed by beta testing among 10 participants [11]. The final version of the intervention was composed of two 15-minute web-based, interactive modules (computerized brief intervention; CBI) followed by three months of daily text messaging. The intervention provided education about increased risk, incorporated content to enhance motivation to avoid alcohol use, and supported the development of personalized coping strategies (e.g., relaxation to manage emotional eating and cravings for alcohol). The intervention was grounded in motivational interviewing (i.e., collaborative, goal-oriented style of communication with an emphasis on strengthening person’s commitment for change) and the content adapted to an individual’s readiness to change [12, 13]. Aligning with the spirit of motivational interviewing, the intervention content did not emphasize abstinence, but rather focused on providing education about the increased risk and included strategies for increasing motivation to avoid alcohol use. A full description of the intervention has been previously published [14]. The final intervention was piloted using a small, randomized controlled trial of 60 participants [15]. Results from the post-intervention assessment suggested that the intervention may reduce likelihood of any drinking as well as amount of drinking. Though the exact timing from re-initiation of alcohol use to development of a postoperative AUD is not yet known, there is evidence to suggest that risk of AUD symptoms begins to increase as patients become more remote from MBS [4]. As such, though there were promising findings at the conclusion of the intervention, it would also be useful to understand whether there are longer-term effects once the intervention ends, as reducing alcohol use long-term may be needed to mitigate risk of a postoperative AUD. The purpose of this study was to examine whether there were longer-term effects following the delivery of a technology-based intervention to reduce alcohol use following MBS.

## Methods

### Participants and Procedures

This study was part of a larger project to develop and evaluate a technology-based intervention to reduce alcohol use after MBS. The study was pre-registered on clinicaltrials.gov (NCT04788316) and a description of the intervention and study protocol was previously published [16]. This project was approved by the health system’s Institutional Review Board.

Patients were contacted about the study if they underwent MBS at a single health system 3–18 months prior to the recruitment date. This eligibility window was selected to target individuals around the time they may re-initiate alcohol use as well as to engage individuals who may be actively drinking at higher amounts [4, 10, 17, 18]. Patients were sent an email that briefly described the study with a link to complete an eligibility survey. Patients were eligible if they had consumed any alcohol prior to having MBS (those without a history of any alcohol use are low risk), had internet access, and had a cell phone with text messaging capabilities. Patients were excluded if they had a history of a preoperative alcohol use disorder. Eligible patients scheduled a time with research staff to learn more about the study and provided informed consent. Participants then completed a baseline assessment through Research Electronic Data Capture (REDCap). After the baseline, participants were randomized to the intervention or a treatment-as-usual control group using urn randomization, stratifying by sex and time since MBS (i.e., < 1 year vs. ≥1 year). Individuals randomized to the intervention received 2 sessions of computerized brief intervention followed by 3-months of daily text messaging. Individuals randomized to the control group received treatment as usual. Prior to MBS, all patients in our program received education about the increased risk of developing a postoperative alcohol use disorder. This program encourages lifelong abstinence from alcohol following MBS. Participants completed measures at baseline and at the 6-month follow-up through REDCap.

### Measures

#### Demographics

Age, sex, race, and type of surgery (Rouxen-Y gastric bypass or sleeve gastrectomy) were reported.

#### Importance to Avoid Alcohol Use

An importance ruler was used to assess motivation to avoid alcohol use at baseline and at the six-month follow-up. Importance rulers are commonly used in motivational interviewing and are a valid method for assessing motivation to change [19]. Participants were asked: “On a scale from 0 (not at all important) to 10 (very important), how important is it to you to avoid any alcohol use?” The 0 to 10 scale captures a wide range of perceptions across two extremes from not at all important to avoid alcohol use to abstinence is very important.

#### Alcohol Use

Alcohol use outcomes included drinking status (i.e., whether alcohol was consumed in the past 30 days), average drinks per drinking day, and frequency of alcohol use were assessed at baseline and the six-month follow-up. To obtain these, items were adapted from the Behavioral Risk Factor Surveillance System Questionnaire [20]. Quantity-frequency measures are the most common method to assess alcohol use and are reliable and valid [21, 22]. Participants were shown an image and description of a standard drink. They were asked to report the number of days they consumed any amount of alcohol in the past 30 days. Participants who reported zero were considered to not have current drinking whereas participants who indicated at least 1 day were categorized as having current drinking. Participants also reported the average number of standard drinks they consumed in a sitting. Alcohol use disorder symptoms were measured with the Alcohol Use Disorder Identification Test - Consumption (AUDIT-C) at baseline and the six-month follow-up [23]. The shorter version was chosen to reduce participant burden while still providing reliable assessment of drinking behavior [23, 24]. Higher scores indicate higher risk for problematic drinking and a score of 3 or greater for women and 4 or greater for men indicates high risk for hazardous drinking.

### Statistical Analyses

Analyses were conducted with SPSS version 29. Descriptive analyses were run for participant characteristics. Following an intention to treat approach, all participants were included in outcome analyses even if they did not complete treatment. Logistic regression was conducted to determine whether the odds of drinking at the 6-month follow-up differed between the intervention and control groups, controlling for baseline drinking status (i.e., whether alcohol was consumed in the 30 days prior to completing the baseline assessment). ANCOVAs were conducted to determine differences at the 6-month follow-up between the intervention and control groups on the importance of avoiding alcohol use, frequency of alcohol use, average number of standard drinks, and AUDIT-C scores controlling for baseline values.

## Results

**Fig. 1:**
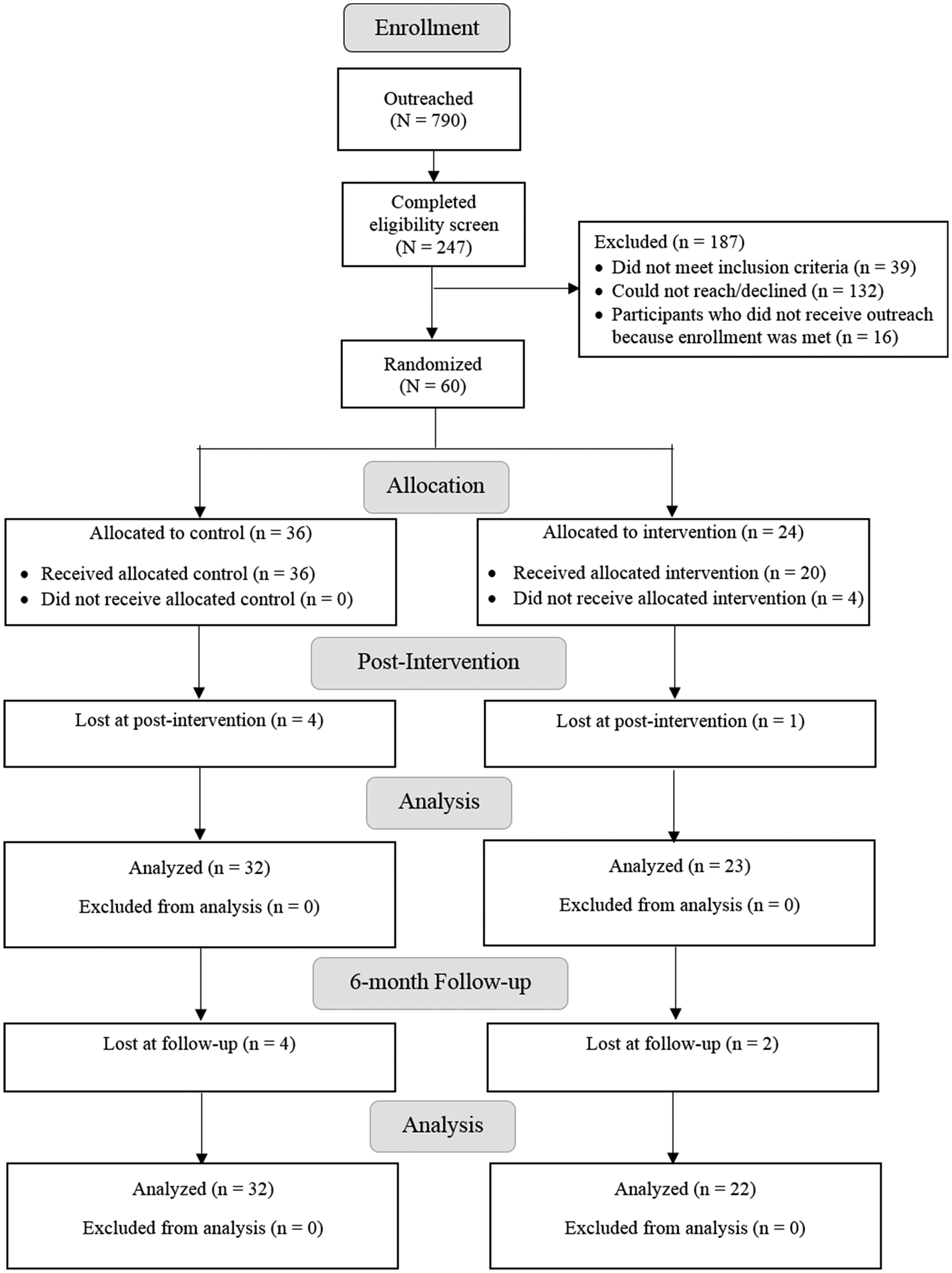
CONSORT diagram of participants in the study

The flow of participants through this study is shown in the CONSORT diagram (Fig. 1). There was 90% (*n* = 54) overall retention at the 6-month follow-up, with 32 participants from the control group (88.9% retention) and 22 participants from the intervention group (91.7% retention). Participant characteristics are presented in Table 1. Participants were primarily female (90.7%), White (55.6%) or Black (38.9%), and had a mean age 44 years. There were no significant baseline differences between the intervention group and control group for age (t= −1.36, *p*= .18), sex (X^2^= 1.51, *p*= .22), race (X^2^= 0.24, *p*= .63), baseline drinking status (X^2^= 0.43, *p*= .51), AUDIT-C scores (t= 0.96, *p*= .34), or amount of preoperative alcohol use (i.e., average number of drinks per sitting; t= 0.62, *p*= .54).

At the 6-month follow-up, the control group had a mean rating of 5.56 (SD = 3.27) on the importance of avoiding alcohol whereas the intervention group had a mean rating of 7.55 (SD = 2.28). However, there was not a significant difference in the importance of avoiding alcohol use between the intervention and control groups at the 6-month follow-up when controlling for baseline ratings (F = 1.48, *p*= .23). The intervention group had lower odds of drinking at the 6-month follow-up compared to the control group, while controlling for baseline drinking status (OR= 0.16, CI: 0.03, 0.88, *p*= .04). There were 22 participants (68.8%) in the control group drinking at the 6-month follow-up compared to 9 participants (40.9%) in the intervention group. There were no between group differences for frequency of alcohol use at the 6-month follow-up (F= 0.40, *p*= .55). The control group had a mean of 4.13 (SD = 6.15) drinking days and the intervention group had a mean of 2.14 (SD = 3.82) drinking days. Participants in the intervention group reported consuming fewer drinks per drinking day compared to the control group at the 6-month follow-up (F = 6.07, *p*= .02). At the 6-month follow-up, the control group consumed an average of 2.09 (SD = 1.69) drinks per drinking day whereas the intervention group consumed an average of 0.95 (SD = 1.40) drinks. There were no significant differences between the intervention and control groups for AUDIT-C scores at the six-month follow-up (F = 2.30, *p*= .14). The control group had a mean score of 2.38 (SD = 2.11) and the intervention group had a mean score of 1.41 (SD = 1.94).

## Discussion

The purpose of this study was to examine whether there were lasting effects of a technology-based intervention to reduce alcohol use among those who underwent MBS after the intervention concluded. Our findings demonstrated that participants in the intervention group were less likely to consume alcohol compared to the control group at the 6-month follow-up. These findings were similar to the outcomes observed at post-intervention, in which individuals randomized to the intervention group were less likely to be consuming alcohol at the conclusion of the intervention [25]. This suggests that the intervention may have had a lasting impact and reduced the likelihood that individuals will consume alcohol following MBS. Moreover, the number of drinks consumed on a drinking day was lower among the intervention group, which was also similar to the findings at post-intervention [26]. This suggests that there could also be a lasting effect on the amount of alcohol consumed. Because the amount of alcohol consumed could lead to a future AUD, preventing re-initiation of post-operative alcohol use and decreasing the amount of alcohol that is consumed could both be effective for mitigating the risk of a future AUD after MBS as well as reduce the harm from heavy drinking [27, 28]. There are other technology-based interventions that have been used to reduce substance use among populations with misuse [29, 30], and this work suggests that a technology-based approach might also be useful as a *prevention* approach.

Despite the positive findings on drinking status and number of drinks, there were no differences between the intervention and control groups for the importance of avoiding alcohol use, frequency of alcohol use, and AUDIT-C scores. These findings were similar to the post-intervention assessment, where there were also no between group differences for these variables. It is possible that the intervention may not have a short- or long-term impact on these variables. One of the a priori hypotheses of this work was that the intervention would increase the rating on the importance of avoiding alcohol use, which would mediate the finding of reduced alcohol use. Because there were no group differences in the importance rating, but there was a difference in drinking status, there may be other mechanisms that play a role in reducing alcohol use. In other patient samples, self-efficacy, perceptions of peer drinking, self-regulation, and coping skills were mechanisms associated with reducing alcohol use [31, 32]. These factors may be important to measure following MBS in future work as well. Alternatively, it is possible that statistically significant differences were not detected due to the small sample size, particularly given that some of the between group differences may be clinically meaningful. As such, it may be worthwhile to continue to examine motivation to avoid alcohol use in future work.

It is important to note several limitations of this study. First, given the pilot nature of this study, there was a small sample size. It is possible that there was not sufficient power to detect group differences. Therefore, it is important to examine the intervention among a larger sample size to more thoroughly understand efficacy of the intervention on outcomes and mechanisms. In addition, participants self-reported their alcohol use, which may not be reliable. Future work should consider using an objective measurement of alcohol use. Finally, the timeframe to measure the final outcomes was at six-months following the conclusion of the intervention. Given that risk for AUD increases as patients become more remote from surgery, longer-term follow-ups are warranted. Nevertheless, there are several strengths of this study as well and these include having a longer-term follow-up of a novel intervention to reduce postoperative alcohol use following MBS and there was a high retention rate.

In summary, there is potential for this intervention to result in reduced alcohol use following MBS and have a continued effect 6 months after the intervention. These findings warrant future study, and a fully powered randomized clinical trial is needed to determine efficacy of this intervention and to ensure that there are lasting effects of the intervention. Future work should also examine whether this intervention reduces risk of developing an AUD. Should this intervention be successful, it may offer a low intensity, low-cost approach to mitigate risk of developing an AUD. In addition, if this intervention is efficacious, it could possibly be adapted to target other types of postoperative substance use as well.

## Figures and Tables

**Table 1:** Participant characteristics (*n* = 54)

	M	SD
Age (yrs)	44.4	10.4
	%	n
Sex		
Female	90.7	49
Male	9.3	5
Race		
White	55.6	30
Black	38.9	21
Other/Multiracial	5.6	3
Surgery Type		
Sleeve gastrectomy	96.3	52
Roux-en-Y gastric bypass	3.7	2
Alcohol use at baseline		
Yes	63.0	34
No	37.0	20

## Data Availability

Data from this study was deposited into the NIMH Data Archive. Data may also be available through request to the corresponding author.

## Key Points

- A technology-based intervention has the potential to result in reduced likelihood of drinking six months after the intervention concludes.
- The intervention may also lead to a lower number of drinks consumed six months after the intervention.
- There were no differences between the intervention and control groups for frequency of drinking six months after the intervention.
